# The usefulness of non-directed bronchoalveolar lavage in diagnosis pneumonia in ICU

**DOI:** 10.1186/2197-425X-3-S1-A711

**Published:** 2015-10-01

**Authors:** T Zawada, J Bartczak, M Kozak, A Wieczorek, P Garba

**Affiliations:** 4 WSK z Poliklinika we Wroclawiu, Wrocław, Poland

## Introduction

Intensive care units are high-risk areas for infections caused by antibiotic-resistant bacteria. Care of ICUs patients involve close contact with hospital staff and provide opportunities for cross-contamination from the environment and from other patients. The resulting colonization of patients is generally accepted as a prerequisite for causing most of nosocomial infections including hospital - aquired pneumonia and VAP. Information about microorganism which cause particular infections and colonization in ICU is essential to prepare local antibiotic guidelines and should be taken into account in implementing empirical treatment.

## Objectives

The aim of the study was to create ICU's microbiological map of pneumonia based on specimens received from non-directed bronchoalveolar lavage.

## Methods

We analyzed the results of the non-directed bronchoalveolar lavage (NBL) collected from patients hospitalized in the ICU during last 5 years. Every patient admitted to the ICU had NBL taken and was categorized to one of three groups: no infection, colonization (colony forming< 10^2^ units/mL), pneumonia (colony forming ≥10^3^ units/mL). We analyzed the types of bacteria which caused colonization or pneumonia and their antibiotic-sensitivity.

## Results

See tables 1, 2 and 3

**Table 1:** Results of the NBL performed on the admission.

	NO INFECTION	COLONIZATION	PNEUMONIA
NUMBER OF PATIENTS (%)	679 (45,5%)	409 (27,5%)	400(27%)

**Table 2:** Identification of microorganisms caused pneumonia.

	% of isolation
S. baumani	31,5%
S. aureus	13,4%
P. aeruginosa	13,1%
E. coli	9,2%
K. pneumoniae	8,6%
C. albicans	7,2%
E. faecalis	5,3%
S.pneumoniae	4,5%
S.oralis, E. Faecium	7,2%

**Table 3:** Antibiotic - sensitivity of A.baumani.

Medicine	susceptible(%)	intermedate (%)	resistant (%)
Collistin	99%	0%	1%
Meropenem	33%	12%	55%
Imipenem	36%	22%	42%
Gentamycin	58%	0%	42%

## Conclusions

The NBL is a useful method to identify infection and colonization of lower airways. It allows to create microbiological map of ICU's residual pathogens and their drug sensitivity, and as a consequents gives intensivist opportunity to implement suitable antibiotic treatment.
